# Supplementary material for: Monthly variance in UK renal transplantation activity: a national retrospective cohort study
Source: BMJ Open. 2019 Sep 17;9(9):e028786. doi: 10.1136/bmjopen-2018-028786 (PMC6756352; doi:10.1136/bmjopen-2018-028786)
Supplement: Author's manuscript [file bmjopen-2018-028786.draft_revisions.pdf]

BMJ Open

BMJ Open is committed to open peer review. As part of this commitment we make the peer review history of every article we publish publicly available.

When an article is published we post the peer reviewers' comments and the authors' responses online. We also post the versions of the paper that were used during peer review. These are the versions that the peer review comments apply to.

The versions of the paper that follow are the versions that were submitted during the peer review process. They are not the versions of record or the final published versions. They should not be cited or distributed as the published version of this manuscript.

BMJ Open is an open access journal and the full, final, typeset and author-corrected version of record of the manuscript is available on our site with no access controls, subscription charges or pay-per-view fees (http://bmjopen.bmj.com).

If you have any questions on BMJ Open's open peer review process please email info.bmjopen@bmj.com

BMJ Open

# Seasonal Variance in Organ Donation and Renal Transplantation in the United Kingdom; Trends and implications for service planning

| | |
|---|---|
| Journal: | *BMJ Open* |
| Manuscript ID | bmjopen-2018-028786 |
| Article Type: | Research |
| Date Submitted by the Author: | 23-Dec-2018 |
| Complete List of Authors: | Lowe, Marcus; Manchester Foundation Trust, Transplantation Laboratory<br>Maidstone, Robert; University of Manchester, Faculty of Biology, Medicine and Health; University of Oxford, Oxford Centre for Diabetes, Endocrinology and Metabolism<br>Poulton, Kay; Manchester University Foundation NHS trust, Transplantation Laboratory<br>Worthington, Judith; Manchester University Foundation NHS trust, Transplantation Laboratory<br>Durrington, Hannah J.; University of Manchester, Faculty of Biology, Medicine and Health; Manchester University Foundation NHS trust, Department of Respiratory Medicine<br>Ray, David; University of Manchester, Faculty of Biology, Medicine and Health; University of Oxford, Oxford Centre for Diabetes, Endocrinology and Metabolism<br>van Dellen, David; University of Manchester, Faculty of Biology, Medicine and Health; Manchester University Foundation NHS trust, Transplant and Endocrine Surgery<br>Asderakis, Argiris; University Hospital of Wales, Cardiff Transplant Unit<br>Blaikley, John; University of Manchester Faculty of Biology, Medicine and Health, ; Manchester University Foundation NHS trust, Medicine<br>Augustine, Titus; University of Manchester, Faculty of Biology, Medicine and Health; Manchester University Foundation NHS trust, Transplant and Endocrine Surgery |
| Keywords: | Renal transplantation < NEPHROLOGY, Seasonal, Healthcare planning |
| | |

SCHOLARONE™
Manuscripts

**Title** Seasonal Variance in Organ Donation and Renal Transplantation in the United Kingdom; Trends and implications for service planning

***Authors***

Marcus P. Lowe[1], Robert Maidstone[2,3], Kay V. Poulton[1], Judith E. Worthington[1], Hannah Durrington[2,4], David W. Ray[2,3], David van Dellen[2,5], Argiris Asderakis[6], John Blaikley[2,4†], Titus Augustine[2,5†]

***Affiliations***

[1]Transplantation Laboratory, Manchester Royal Infirmary, Manchester University NHS Foundation Trust

[2]Faculty of Biology, Medicine and Health, University of Manchester, Manchester, UK

[3]Oxford Centre for Diabetes, Endocrinology and Metabolism, University of Oxford, Oxford and NIHR Oxford Biomedical Research Centre, John Radcliffe Hospital OX3 9DU. UK

[4]Department of Respiratory Medicine, Manchester University NHS Foundation Trust, Manchester, UK

[5]Transplant and Endocrine Surgery, Manchester University NHS Foundation Trust, Manchester, UK

[4]Department of Cardiothoracic Medicine and Transplantation, Manchester University NHS Foundation Trust

[6]Cardiff Transplant Unit, University Hospital of Wales, Cardiff

**† = joint corresponding authors**

**Funding:** JB holds a MRC clinician scientist award (MR/L006499/1). DWR holds a Wellcome Trust investigator award (107851/Z/15/Z). HD is supported by an Asthma UK Senior Clinical Academic Development Award (AUK-SCAD-2013-229).

For peer review only

**Acknowledgement:** The authors would like to acknowledge Phil Foden for his contribution to the statistically analysis of the results, the NHSBT and U.K. renal transplant centers for collecting and providing the data.

Keywords: Renal transplantation, Seasonal Variation, Healthcare Planning

Word Count: 1,680

For peer review only

**Strengths and limitations of the study**

- The study was a 10 year retrospective study involving all the registered renal transplant recipients in the UK over this time period.
- The database is filled in by each transplant center; therefore the data has not been independently corroborated.
- A minority of patients will have refused to be enrolled in the national database potentially affecting our findings.
- The database is used to audit transplant provision in the UK therefore it was not set up specifically for this research project

For peer review only

**Abstract**

**Objective:** To identify whether renal transplant activity varies in a reproducible manner across the year

**Design:** Retrospective cohort study using NHS blood and transplant (NHSBT) data

**Setting:** All renal transplant centers in the UK

**Participants:** 24,270 patients who underwent renal transplantation between 2005 and 2014

**Primary outcome:** Monthly transplant activity was analysed to see if transplant activity showed variation during the year

**Secondary Outcome:** The number of organs rejected due to healthcare capacity was analysed to see if this affected transplantation rates.

**Results:** Analysis of national transplant data revealed a reproducible yearly variance in transplant activity. This activity increased in late autumn and early winter (p=0.05) and could be attributed to increased rates of living (October-November) and deceased organ donation (November, December). An increase in deceased donation was attributed to a rise in donors following cerebrovascular accidents and hypoxic brain injury. Other causes of death (infections and road traffic accidents) were more seasonal in nature peaking in the winter or summer respectively. Only 1.4% of transplants to intended recipients were redirected due to a lack of healthcare capacity, suggesting that capacity pressures in the NHS did not significantly affect transplant activity.

**Conclusion:** UK renal transplant activity peaks in late autumn/ winter in contrast to other countries. Currently healthcare capacity does not affect transplant activity;

For peer review only

however this may change if transplantation activity increases as the spike in transplant activity coincides with peak activity in the national healthcare system.

For peer review only

# Introduction

When organ donors and families of potential donors consent to donation, they make a very valuable gift which is life changing for the recipient. Despite recent improvements in the United Kingdom (UK)[1] and other countries regarding access to transplantation demand for renal transplantation exceeds the number of available donors leading to increased patient mortality and morbidity. Since many potential donors cannot be utilised for various reasons it is vital that these are minimised so that every potential organ for transplant is utilised for the primary intended recipient. One of the reasons transplantation does not proceed is due to a lack of clinical capacity. Therefore healthcare planning plays a key role in ensuring that sufficient capacity exists so that all transplants are utilised for the primary intended recipients. This could be potentially difficult as the NHS often works near or at maximum capacity[2], especially in winter months.

Renal transplantation uses both living and deceased donors. Human mortality rates are known to oscillate in a seasonal manner from some diseases[3]. This can be attributed to the effects of endogenous seasonal rhythms and climatic factors on human performance and activity patterns. In the U.K. seasonal variations are commonly observed for infectious diseases such as influenza[4], cerebrovascular disease[5-10] and myocardial infarctions[11]. Several studies have recently shown how understanding these oscillations are crucial for planning and delivering optimum health care delivery[12]. Despite the USA covering many different climate zones, cerebrovascular accidents in the USA peak in the winter [13], in a similar pattern to the UK [5-10]. Surprisingly despite these deaths being a leading cause of organ donation

For peer review only

transplant activity in the USA is lowest in the winter months for both renal[14] and heart transplantation[15]. A similar pattern is also seen in Italy[16], suggesting this seasonal variance could be observed in other countries.

To the best of our knowledge seasonal fluctuations in transplant activity have not been investigated in the UK. We therefore examined the national cohort over a 10 year period to establish both seasonal fluctuations and whether healthcare capacity influences activity.

For peer review only

# Methods

All UK renal adults transplants, performed between 2005 and 2014 to recipients over 18 years old, were included in the study. Data was provided by NHSBT (NHS Blood and Transplant) who maintain a comprehensive national database on the 24 adult kidney transplant centres in the United Kingdom. This data was combined with data collected on donor activity, provided by the National Organ Retrieval Service (NORS). Deceased donation is categorised into donation after brain death (DBD) and donation after circulatory death DCD). All donor deaths are further classified according to the cause of mortality. “Cerebrovascular event” was defined as death due to intracranial haemorrhage, intracranial thrombus or unclassified intracranial event. “Infection” was defined as death due to meningitis, septicaemia, pneumonia or unclassified infection. Deceased donor transplants form about two thirds of all UK transplant activity. The other one third of kidney transplants, are transplants from living donors. Living donor transplants are logistically different as they are planned and scheduled elective cases depending on several factors which can usually be controlled. Deceased donor transplants on the other hand are unplanned occurring when organs become available after death of a suitable donor. Recipients are then allocated organs according to agreed national allocation criteria.

Data was analysed according to the month and year the transplant occurred. Donation details were analysed in a similar manner. The observed data was compared to the expected transplant activity calculated by measuring the total number of transplants divided by the number of days in the same year.

Data was also analysed according to organs offered to different centres for named patients and declined, for various reasons by the centre. Organs are declined for various clinical reasons and, anecdotally, rarely due to capacity and peaks of activity.

For peer review only

Capacity issues for the purposes of this paper were sub classified into different categorical values (centre already transplanting, no beds, no staff, no theatre, no time).

***Statistical methods***

Generalised linear models (GLMs), with a Poisson link function were used to model the effect that month of the year had on our data. Offset variables were used to account for the slight differences in number of days per month. To account for the unknown correlation structure in our data, generalised estimating equations (GEEs) were used to estimate the GLM parameters[17].

Chi-squared goodness of fit tests were used to assess whether the observed number of transplants per month differed from the expected value under the GLM. When this test was significant (i.e. the expected values were significantly different to the observed values), Pearson residuals were used to identify the particular months which caused this mismatch. Residuals that are greater than 2 in magnitude suggest some degree of lack of fit[18].

Sine and cosine curves were fitted to the transplant data to investigate whether repeating patterns occurred across the months. All analyses were performed using SPSS 22 (IBM corp)

**Ethics:**

This study received ethical permission from NHSBT

For peer review only

# Results

During the ten-year study period 24,270 adult kidney transplants were performed in the UK. 15,094 (62%) were from deceased donors and 9,166 (38%) were from living donors

### Kidney Transplant activity changes in a consistent manner throughout the year

Kidney Transplant activity varied by 17.1% within a year (p<0.01, chi-square, fig 1a). Transplant activity was highest in November (11% increase from mean, p<0.01) and lowest in August (6% decrease from mean, p<0.01). Investigating seasonal variation, transplant activity increased in the autumn compared to spring and summer (p<0.05 one way ANOVA); these patterns were consistent every year during the 10 year study period (p<0.01 GEE).

Kidney transplantation utilises both living and deceased donors. Our dataset was therefore examined to see if one of these types was responsible for the variance in activity. Transplant activity utilising organs from living donors (fig. 1b) significantly increased in October and November and decreased in December, April, May and August. Transplant activity utilising organs from deceased donors (fig. 1c) increased in December and a similar trend was observed in November. Therefore the increase in activity for November, which has the highest activity, is due to an increase in transplants utilising kidneys from both living and deceased organ donors. This contrasts with August, which had the lowest activity, and is solely explained by a fall in living donor activity.

### Incidence for brain injury donors fluctuates over the year

For peer review only

Donation from cerebrovascular and hypoxic brain injury accounted for 78.8% of all deceased donor transplants, therefore we hypothesised that the spike in deceased donor transplants is due to an increase in donors from these categories during November and December. Cerebrovascular deaths, as a cause of donor death, were significantly increased in November (12% increase from the mean, $p<0.01$) (fig. 2a). Donations from donors who had experienced a hypoxic brain injury tended to increase during this period with the highest spike being in December (25% increase from the mean, $p=0.15$) (fig 2b). When these results are combined, they mirror the fluctuations seen for all deceased organ transplant activity (fig. 2c) with signifcant elevations in transplant activity during November and December ($p<0.01$).

**Seasonal Variance in the incidence of donors after infection**

We also examined other causes of donor death which significantly varied over the year (table 1). Donations from donors dying from infective causes peaked in the winter months and declined in the summer (fig3b) this relationship could be explained by a cosine curve (fig 3b, $p<0.05$) suggesting an underlying seasonal oscillation. Donations after road traffic accidents (fig. 3a) were higher during late spring, summer and early autumn (April-September) compared to other times of the year.

**The effect of healthcare capacity on transplant activity**

The U.K. healthcare system routinely works at high capacity[19], potentially resulting in reduced transplantation rates due to bed and staffing shortages. We therefore examined whether the reproducible monthly changes in transplant activity could be attributed to a lack of capacity in the healthcare system. Although a large number 28,789 of deceased donor offers were declined for transplantation over the study

For peer review only

period only a small fraction of these were due to lack of capacity (n=480 mean 1.22% (±0.22%SD).

For peer review only

### *Discussion*

The National Health Service in the U.K. is coping with increased pressures especially in winter[19]. This study reveals that UK kidney transplant activity peaks in November and December, at the beginning of winter, in contrast with a number of European[16 20] and North American centres[14 15]. This unexpected finding is due to increased living and deceased donation. Although detailed causal analysis was beyond the scope of the study, the increases in deceased donation could be attributed to both increases in cerebrovascular and hypoxic brain events. This is consistent with findings from previous studies studying the incidence of these events both in the UK[7] and other northern hemisphere countries[9 10].

The winter surge in transplant activity has important implications for the U.K. health system (NHS). During the UK winter there is also a surge in emergency admissions to hospitals, placing the system under significant strain, sometimes resulting in cancellation of elective and semi-elective operations for up to several months[21]. It was therefore reassuring that we found no solid evidence of transplant surgery utilising deceased donors being cancelled during the winter months. Despite this however it is important that transplant departments plan for this predictable and reproducible surge in transplant activity. We are confident that this is likely to continue into the future as two different statistical tests (chi-square and GEE) produced similar results. Healthcare capacity planning should take our findings into account when planning capacity at transplant centres. This is especially important as UK transplant activity should significantly increase the next two years.

For peer review only

Certain limitations should be noted when interpreting the results of this study. We were not able to investigate whether healthcare capacity influenced the number of living donations since this data was not recorded. Since this was a retrospective study there is always the potential for inherent bias, although the large dataset will minimise this effect.

Our study clearly shows that UK renal transplant activity increases in the winter in contrast to previous studies investigating seasonal transplant activity in other countries. This could have implications for the UK health system since winter is when the British health system is placed under maximal strain and therefore should be taken into account for any future planning of increased resources and staffing.

**Authorship:** AT and JB conceived the study. AT, JB and ML obtained and analysed the data. RM did the statistical analysis. KP, JW, HD, DD, DWR, HD, AA substantially contributed to the interpretation of the data. AT, JB and AA drafted the manuscript, with the other authors revising it critically. .

**Conflicts of Interest:** The authors declare no conflicts of interest

For peer review only

# References

1. Transplant NBa. Organ Donation and Transplantation Activity report 2017/18, 2018.
2. Scriven DN. Capacity issues need to be tackled by looking at all of acute provision. *British Journal of Hospital Medicine* 2018;79(6):304-05. doi: 10.12968/hmed.2018.79.6.304
3. Rolden HJ, Rohling JH, van Bodegom D, et al. Seasonal Variation in Mortality, Medical Care Expenditure and Institutionalization in Older People: Evidence from a Dutch Cohort of Older Health Insurance Clients. *PloS one* 2015;10(11):e0143154. doi: 10.1371/journal.pone.0143154 [published Online First: 2015/11/17]
4. Paules C, Subbarao K. Influenza. *Lancet (London, England)* 2017 doi: 10.1016/s0140-6736(17)30129-0 [published Online First: 2017/03/18]
5. Anlar O, Tombul T, Unal O, et al. Seasonal and environmental temperature variation in the occurrence of ischemic strokes and intracerebral hemorrhages in a Turkish adult population. *The International journal of neuroscience* 2002;112(8):959-63. [published Online First: 2002/11/27]
6. de Steenhuijsen Piters WA, Algra A, van den Broek MF, et al. Seasonal and meteorological determinants of aneurysmal subarachnoid hemorrhage: a systematic review and meta-analysis. *Journal of neurology* 2013;260(2):614-9. doi: 10.1007/s00415-012-6687-z [published Online First: 2012/10/17]
7. Haberman S, Capildeo R, Rose FC. The seasonal variation in mortality from cerebrovascular disease. *Journal of the neurological sciences* 1981;52(1):25-36. [published Online First: 1981/10/01]
8. Mao Y, Schnytzer Y, Busija L, et al. "MOONSTROKE": Lunar patterns of stroke occurrence combined with circadian and seasonal rhythmicity--A hospital based study. *Chronobiology international* 2015;32(7):881-8. doi: 10.3109/07420528.2015.1049614 [published Online First: 2015/07/15]
9. Sipila JO, Ruuskanen JO, Kauko T, et al. Seasonality of stroke in Finland. *Annals of medicine* 2017;49(4):310-18. doi: 10.1080/07853890.2016.1254350 [published Online First: 2016/10/28]
10. Telman G, Sviri GE, Sprecher E, et al. Seasonal variation in spontaneous intracerebral hemorrhage in northern Israel. *Chronobiology international* 2017;34(5):563-70. doi: 10.1080/07420528.2016.1278223 [published Online First: 2017/02/06]
11. Yang L, Li L, Lewington S, et al. Outdoor temperature, blood pressure, and cardiovascular disease mortality among 23 000 individuals with diagnosed cardiovascular diseases from China. *European heart journal* 2015;36(19):1178-85. doi: 10.1093/eurheartj/ehv023 [published Online First: 2015/02/19]
12. Stevenson TJ, Visser ME, Arnold W, et al. Disrupted seasonal biology impacts health, food security and ecosystems. *Proceedings Biological sciences* 2015;282(1817):20151453. doi: 10.1098/rspb.2015.1453 [published Online First: 2015/10/16]
13. Kalkstein AJ. Regional similarities in seasonal mortality across the United States: an examination of 28 metropolitan statistical areas. *PloS one* 2013;8(5):e63971. doi: 10.1371/journal.pone.0063971 [published Online First: 2013/06/05]

For peer review only

14. Rosendale JD. Organ donation in the United States: 1988-2003. *Clinical transplants* 2004:41-50. [published Online First: 2006/05/18]
15. Grodin JL, Ayers CR, Thibodeau JT, et al. Variation of heart transplant rates in the United States during holidays. *Clinical transplantation* 2014;28(8):877-82. doi: 10.1111/ctr.12396 [published Online First: 2014/06/17]
16. Santori G, Fontana I, Bertocchi M, et al. A time series analysis performed on a 25-year period of kidney transplantation activity in a single center. *Transplantation proceedings* 2010;42(4):1080-3. doi: 10.1016/j.transproceed.2010.03.036 [published Online First: 2010/06/11]
17. Liang K-Y, Zeger SL. Longitudinal data analysis using generalized linear models. *Biometrika* 1986;73(1):13-22. doi: 10.1093/biomet/73.1.13
18. Menard S. Applied Logistic Regression Analysis. 2nd ed. USA: Sage 2002.
19. Iacobucci G. NHS "winter" pressure could become norm in summer, experts warn. *BMJ (Clinical research ed)* 2016;352:i905. doi: 10.1136/bmj.i905 [published Online First: 2016/02/14]
20. Leal J, Caballero F, Garcia-Sousa S, et al. Distribution of organ donors on a weekly and annual basis according to cause of death. *Transplantation proceedings* 1999;31(6):2602-3. [published Online First: 1999/09/29]
21. Iacobucci G. NHS cancels planned surgery and outpatient appointments in response to winter crisis. *BMJ (Clinical research ed)* 2018;360 doi: 10.1136/bmj.k19

For peer review only

# Tables

***Table 1***

| **Cause of death in the Donor** | **n** | **p value for whether donation varied across the year** |
|---|---|---|
| Intracranial / Cerebrovascular causes | 9452 | *<0.01* |
| Hypoxic brain damage | 2448 | *<0.01* |
| Trauma | 1532 | *<0.01* |
| Infective Causes | 367 | *<0.01* |
| Cardiovascular | 237 | 0.13 |
| Respiratory / Pulmonary causes | 192 | 0.17 |
| Brain Tumors | 180 | 0.14 |
| Poisoning / Drug overdose | 55 | 0.03 |
| Organ Failure (Various Causes) | 53 | *<0.01* |
| Other / Unknown | 578 | *0.01* |

**Table 1: Seasonal variation in kidney transplants utilising organs from deceased donors.** All deceased donations over a 10 year period were split into groups according to aetiology, defined by NHSBT. The incidence for the majority (7/10) of causes varied significantly during the year.

For peer review only

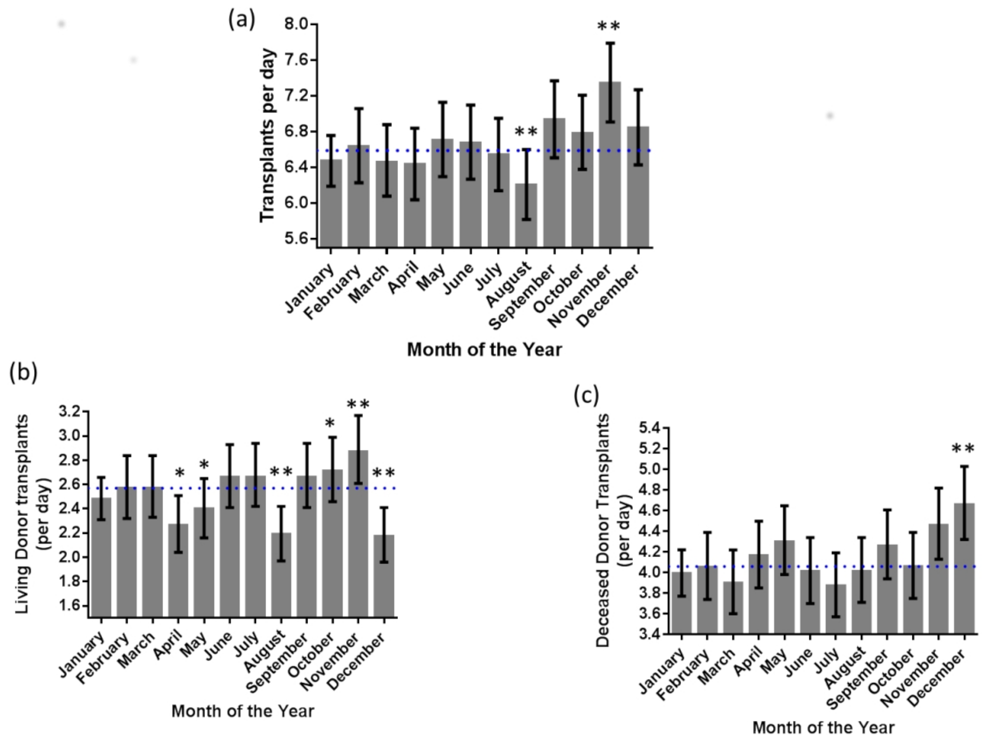


Figure 1: Transplant activity changes throughout the year. The number of transplants per day (a) varied significantly throughout the year (p<0.01). The November peak in overall transplant activity can be attributed to an increase in transplant activity from both living (b) and deceased donors (c). (*=p<0.05, **=p<0.01 Pearson residual)

123x93mm (300 x 300 DPI)

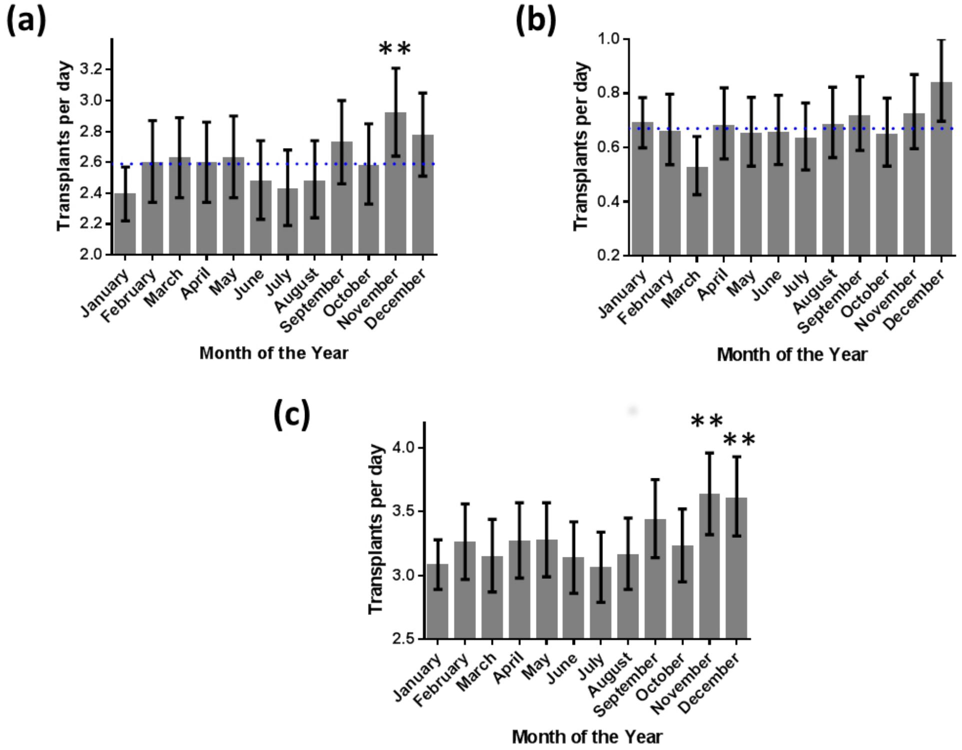


Figure 2: Fluctuations in the number of donors with cerebrovascular and hypoxic brain injury explains the fluctuations in deceased donors. Organ donation after cerebrovascular events (a) were elevated in September, November and December. Donation after hypoxic brain damage (b) showed a similar end of year increase in activity. When both these causes were combined (c) it closely mirrored the fluctuation seen in transplant activity for all deceased donors (shown in fig 1c).

86x69mm (300 x 300 DPI)

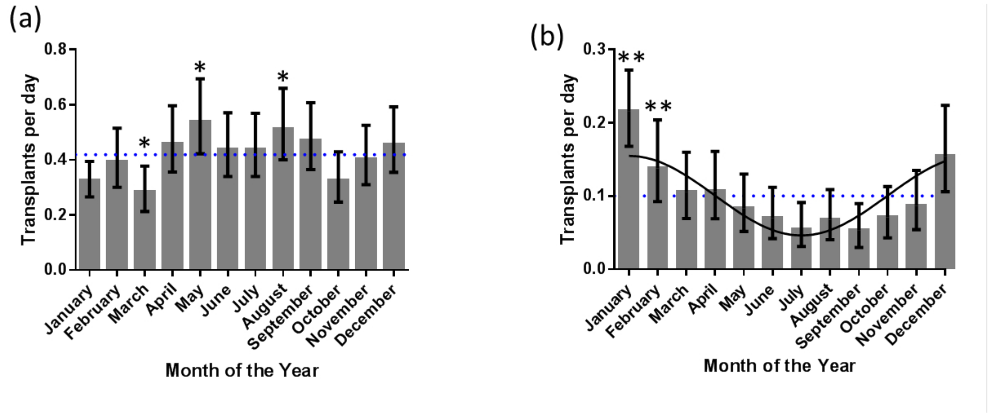


Figure 3: Donation from donors with infection and trauma also oscillate throughout the year. The rates of donation after infection and trauma were plotted against months of the year. Donation rates after trauma increased in the summer as opposed to the winter (a). For infection (b) the rates oscillated (cosine p<0.05) throughout the year peaking in the winter months.

103x44mm (300 x 300 DPI)

# Reporting checklist for cohort study.

Based on the STROBE cohort guidelines.

## Instructions to authors

Complete this checklist by entering the page numbers from your manuscript where readers will find each of the items listed below.

Your article may not currently address all the items on the checklist. Please modify your text to include the missing information. If you are certain that an item does not apply, please write "n/a" and provide a short explanation.

Upload your completed checklist as an extra file when you submit to a journal.

In your methods section, say that you used the STROBE cohort reporting guidelines, and cite them as:

von Elm E, Altman DG, Egger M, Pocock SJ, Gotzsche PC, Vandenbroucke JP. The Strengthening the Reporting of Observational Studies in Epidemiology (STROBE) Statement: guidelines for reporting observational studies.

For peer review only

| | | Reporting Item | Page Number |
|---|---|---|---|
| Title | #1a | Indicate the study’s design with a commonly used term in the title or the abstract | 1 |
| Abstract | #1b | Provide in the abstract an informative and balanced summary of what was done and what was found | 3 |
| Background / rationale | #2 | Explain the scientific background and rationale for the investigation being reported | 4 |
| Objectives | #3 | State specific objectives, including any prespecified hypotheses | 4 |
| Study design | #4 | Present key elements of study design early in the paper | 6 |
| Setting | #5 | Describe the setting, locations, and relevant dates, including periods of recruitment, exposure, follow-up, and data collection | 6 |
| Eligibility criteria | #6a | Give the eligibility criteria, and the sources and methods of selection of participants. Describe methods of follow-up. | 6 |
| | #6b | For matched studies, give matching criteria and number of exposed and | n/a |

| | | | |
|---|---|---|---|
| | | unexposed | |
| Variables | #7 | Clearly define all outcomes, exposures, predictors, potential confounders, and effect modifiers. Give diagnostic criteria, if applicable | 6 and 7 |
| Data sources / measurement | #8 | For each variable of interest give sources of data and details of methods of assessment (measurement). Describe comparability of assessment methods if there is more than one group. Give information separately for for exposed and unexposed groups if applicable. | 6 |
| Bias | #9 | Describe any efforts to address potential sources of bias | n/a |
| Study size | #10 | Explain how the study size was arrived at | 6 |
| Quantitative variables | #11 | Explain how quantitative variables were handled in the analyses. If applicable, describe which groupings were chosen, and why | 6 and 7 |
| Statistical methods | #12a | Describe all statistical methods, including those used to control for confounding | 7 |
| | #12b | Describe any methods used to examine subgroups and interactions | 7 |
| | #12c | Explain how missing data were addressed | n/a |
| | #12d | If applicable, explain how loss to follow-up was addressed | n/a |
| | #12e | Describe any sensitivity analyses | n/a |
| Participants | #13a | Report numbers of individuals at each stage of study—eg numbers potentially eligible, examined for eligibility, confirmed eligible, included in the study, completing follow-up, and analysed. Give information separately for for exposed and unexposed groups if applicable. | n/a |
| | #13b | Give reasons for non-participation at each stage | n/a |
| | #13c | Consider use of a flow diagram | n/a |
| Descriptive data | #14a | Give characteristics of study participants (eg demographic, clinical, social) and information on exposures and potential confounders. Give information separately for exposed and unexposed groups if applicable. | 10 |
| | #14b | Indicate number of participants with missing data for each variable of interest | 10 |
| | #14c | Summarise follow-up time (eg, average and total amount) | n/a |

For peer review only

| | | | |
|---|---|---|---|
| Outcome data | #15 | Report numbers of outcome events or summary measures over time. Give information separately for exposed and unexposed groups if applicable. | 10 |
| Main results | #16a | Give unadjusted estimates and, if applicable, confounder-adjusted estimates and their precision (eg, 95% confidence interval). Make clear which confounders were adjusted for and why they were included | 10-12 |
| | #16b | Report category boundaries when continuous variables were categorized | n/a |
| | #16c | If relevant, consider translating estimates of relative risk into absolute risk for a meaningful time period | n/a |
| Other analyses | #17 | Report other analyses done—e.g., analyses of subgroups and interactions, and sensitivity analyses | 10-12 |
| Key results | #18 | Summarise key results with reference to study objectives | 13-14 |
| Limitations | #19 | Discuss limitations of the study, taking into account sources of potential bias or imprecision. Discuss both direction and magnitude of any potential bias. | 13-14 |
| Interpretation | #20 | Give a cautious overall interpretation considering objectives, limitations, multiplicity of analyses, results from similar studies, and other relevant evidence. | 13-14 |
| Generalisability | #21 | Discuss the generalisability (external validity) of the study results | 13-14 |
| Funding | #22 | Give the source of funding and the role of the funders for the present study and, if applicable, for the original study on which the present article is based | 1 |

The STROBE checklist is distributed under the terms of the Creative Commons Attribution License CC-BY. This checklist was completed on 30. November 2018 using http://www.goodreports.org/, a tool made by the EQUATOR Network in collaboration with Penelope.ai

For peer review only

BMJ Open

# Seasonal Variance in Organ Donation and Renal Transplantation in the United Kingdom; Trends and implications for service planning

| | |
|---|---|
| Journal: | *BMJ Open* |
| Manuscript ID | bmjopen-2018-028786.R1 |
| Article Type: | Research |
| Date Submitted by the Author: | 23-May-2019 |
| Complete List of Authors: | Lowe, Marcus; Manchester Foundation Trust, Transplantation Laboratory<br>Maidstone, Robert; University of Manchester, Faculty of Biology, Medicine and Health; University of Oxford, Oxford Centre for Diabetes, Endocrinology and Metabolism<br>Poulton, Kay; Manchester University Foundation NHS trust, Transplantation Laboratory<br>Worthington, Judith; Manchester University Foundation NHS trust, Transplantation Laboratory<br>Durrington, Hannah J.; University of Manchester, Faculty of Biology, Medicine and Health; Manchester University Foundation NHS trust, Department of Respiratory Medicine<br>Ray, David; John Radcliffe Hospital, NIHR Oxford Biomedical Research Centre; University of Oxford, Oxford Centre for Diabetes, Endocrinology and Metabolism<br>van Dellen, David; University of Manchester, Faculty of Biology, Medicine and Health; Manchester University Foundation NHS trust, Transplant and Endocrine Surgery<br>Asderakis, Argiris; University Hospital of Wales, Cardiff Transplant Unit<br>Blaikley, John; University of Manchester Faculty of Biology, Medicine and Health, ; Manchester University Foundation NHS trust, Medicine<br>Augustine, Titus; University of Manchester, Faculty of Biology, Medicine and Health; Manchester University Foundation NHS trust, Transplant and Endocrine Surgery |
| <b>Primary Subject Heading</b>: | Renal medicine |
| Secondary Subject Heading: | Renal medicine |
| Keywords: | Renal transplantation < NEPHROLOGY, Seasonal, Healthcare planning |
| | |

SCHOLARONE™
Manuscripts

**Title** Seasonal Variance in Organ Donation and Renal Transplantation in the United Kingdom; Trends and implications for service planning

***Authors***

Marcus P. Lowe[1], Robert Maidstone[2,3], Kay V. Poulton[1], Judith E. Worthington[1], Hannah Durrington[2,4], David W. Ray[3,5], David van Dellen[2,6], Argiris Asderakis[7], John Blaikley[2,8†], Titus Augustine[2,6†]

***Affiliations***

[1]Transplantation Laboratory, Manchester Royal Infirmary, Manchester University NHS Foundation Trust

[2]Faculty of Biology, Medicine and Health, University of Manchester, Manchester, UK

[3]Oxford Centre for Diabetes, Endocrinology and Metabolism, University of Oxford, OX37LE

[4]Department of Respiratory Medicine, Manchester University NHS Foundation Trust, Manchester, UK

[5]NIHR Oxford Biomedical Research Centre, John Radcliffe Hospital OX3 9DU

[6]Transplant and Endocrine Surgery, Manchester University NHS Foundation Trust, Manchester, UK

[7]Cardiff Transplant Unit, University Hospital of Wales, Cardiff

[8]Department of Cardiothoracic Medicine and Transplantation, Manchester University NHS Foundation Trust

**†= joint corresponding authors**

**Correspondence can be addressed to: titus.augustine@mft.nhs.uk or john.blaikley@manchester.ac.uk**

For peer review only

Keywords: Renal transplantation, Seasonal Variation, Healthcare Planning

Word Count: 1,918

For peer review only

## Abstract

**Objective:** To identify whether renal transplant activity varies in a reproducible manner across the year

**Design:** Retrospective cohort study using NHS blood and transplant (NHSBT) data

**Setting:** All renal transplant centers in the UK

**Participants:** 24,270 patients who underwent renal transplantation between 2005 and 2014

**Primary outcome:** Monthly transplant activity was analysed to see if transplant activity showed variation during the year

**Secondary Outcome:** The number of organs rejected due to healthcare capacity was analysed to see if this affected transplantation rates.

**Results:** Analysis of national transplant data revealed a reproducible yearly variance in transplant activity. This activity increased in late autumn and early winter ($p=0.05$) and could be attributed to increased rates of living (October-November) and deceased organ donation (November, December). An increase in deceased donation was attributed to a rise in donors following cerebrovascular accidents and hypoxic brain injury. Other causes of death (infections and road traffic accidents) were more seasonal in nature peaking in the winter or summer respectively. Only 1.4% of transplants to intended recipients were redirected due to a lack of healthcare capacity, suggesting that capacity pressures in the NHS did not significantly affect transplant activity.

**Conclusion:** UK renal transplant activity peaks in late autumn/ winter in contrast to other countries. Currently healthcare capacity, though under strain, does not affect transplant activity; however this may change if transplantation activity increases in line


For peer review only

with national strategies as the spike in transplant activity coincides with peak activity in the national healthcare system.

For peer review only

**Strengths and limitations of the study**

- The study was a 10-year retrospective study involving all the registered renal transplant recipients in the UK over this time period.

- The national transplant database is filled using data submitted by each transplant center therefore, the data has not been independently corroborated.

- A minority of patients will have refused to be enrolled in the national database potentially affecting our findings.

- The database is used to audit transplant provision and outcomes in the UK therefore it was not set up specifically for this research project

For peer review only

## Introduction

When organ donors and families of potential donors consent to donation, they make a very valuable gift which is life changing for the recipient. Despite recent improvements in the United Kingdom (UK)[1] and other countries regarding access to transplantation, demand for renal transplantation still exceeds the number of available donors[2]. This results in increased patient mortality and morbidity[3]. Since many potential donors cannot be utilised for various reasons, it is vital that these reasons are minimised so that every potential organ for transplant is utilised for the primary intended recipient. One of the reasons transplantation does not proceed is due to a lack of clinical capacity. Therefore healthcare planning plays a key role in ensuring that sufficient capacity exists so that all transplants are utilised for the primary intended recipients. This could be potentially difficult as the NHS often works near or at maximum capacity[4], especially in winter months.

Renal transplantation uses both living and deceased donors. Human mortality rates are known to oscillate in a seasonal manner from some diseases[5]. This can be attributed to the effects of endogenous seasonal rhythms and climatic factors on human performance and activity patterns. In the U.K. seasonal variations are commonly observed for infectious diseases such as influenza[6], cerebrovascular disease[7-12] and myocardial infarctions[13]. Several studies have recently shown how understanding these oscillations are crucial for planning and delivering optimum health care delivery[14]. Despite the USA covering many different climate zones, cerebrovascular accidents in the USA peak in the winter [15], in a similar pattern to the UK [7-12]. Surprisingly despite these deaths being a leading cause of organ donation transplant activity in the USA is lowest in the winter months for both renal[16] and heart

For peer review only

transplantation[17]. A similar pattern is also seen in Italy[18], suggesting this seasonal pattern is conserved in other countries.

To the best of our knowledge seasonal fluctuations in transplant activity have not been investigated in the UK. We therefore examined the national cohort over a 10-year period to establish both seasonal fluctuations and whether healthcare capacity influences activity.

For peer review only

# Methods

All UK renal adult transplants, performed between 2005 and 2014 to recipients over 18 years old, were included in the study. Data was provided by NHSBT (NHS Blood and Transplant) who maintain a comprehensive national database on the 24 adult kidney transplant centres in the United Kingdom. This data was combined with data collected on donor activity, provided by the National Organ Retrieval Service (NORS). Deceased donation is categorised into donation after brain death (DBD) and donation after circulatory death DCD). All donor deaths are further classified according to the cause of mortality. “Cerebrovascular event” was defined as death due to intracranial haemorrhage, intracranial thrombus or unclassified intracranial event. “Infection” was defined as death due to meningitis, septicaemia, pneumonia or unclassified infection. Deceased donor transplants form about two thirds of all UK kidney transplant activity. The other one third of kidney transplants are transplants from living donors. Living donor transplants are logistically different as they are planned and scheduled elective cases depending on several factors which can usually be controlled. Deceased donor transplants on the other hand are unplanned occurring when organs become available after death of a suitable donor. Recipients are then allocated organs according to agreed national allocation criteria.

Data was analysed according to the month and year the transplant occurred. Donation details were analysed in a similar manner. The observed data was compared to the expected transplant activity calculated by measuring the total number of transplants divided by the number of days in the same year.

Data was also analysed according to organs offered to different centres for named patients and declined, for various reasons by the centre. Organs are declined for various clinical reasons and, anecdotally, rarely due to capacity and peaks of activity.

For peer review only

Capacity issues for the purposes of this paper were sub classified into different categorical values (centre already transplanting, no beds, no staff, no theatre, no time).

### *Statistical methods*

Generalised linear models (GLMs), with a Poisson link function were used to model the effect that month of the year had on our data. Offset variables were used to account for the slight differences in number of days per month. To account for the unknown correlation structure in our data, generalised estimating equations (GEEs) per year were used to estimate the GLM parameters[19].

Chi-squared goodness of fit tests assessed whether the observed number of transplants per month differed from the expected value if there was no seasonal variability. When this test was significant (i.e. the expected values were significantly different to the observed values), Pearson residuals were used to identify the particular months which caused this mismatch. Residuals that are greater than 2 in magnitude suggest some degree of lack of fit[20].

Sine and cosine curves were fitted to the transplant data to investigate whether repeating patterns occurred across the months. All analyses were performed using SPSS 22 (IBM corp)

**Patient and Public Involvement:**

Patients and Public were not involved in the design or analysis of data for this study

**Ethics:**

Data was collected by NHSBT and analysed after obtaining approval from them in accordance with their policies

**Data Availability:**

For peer review only

Raw data will be available from the corresponding author after permission has been obtained from NHSBT. The statistical code has been uploaded onto a data repository DOI: 10.17632/48nxwvcfnh.1

For peer review only

# Results

During the ten-year study period 24,270 adult kidney transplants were performed in the UK. 15,094 (62%) were from deceased donors and 9,166 (38%) were from living donors

**Kidney Transplant activity changes in a consistent manner throughout the year**

Kidney Transplant activity varied by 17.1% within a year ($p<0.01$, chi-square, fig 1a). Transplant activity was highest in November (11% increase from mean, $p<0.01$) and lowest in August (6% decrease from mean, $p<0.01$). Investigating seasonal variation, transplant activity increased in the autumn compared to spring and summer ($p<0.05$ one-way ANOVA); these patterns were consistent every year during the 10 year study period ($p<0.01$ GEE).

Kidney transplantation utilises both living and deceased donors. Our dataset was therefore examined to see if one of these types was responsible for the variance in activity. Transplant activity utilising organs from living donors (fig. 1b) significantly increased in October and November and decreased in December, April, May and August. Transplant activity utilising organs from deceased donors (fig. 1c) increased in December and a similar trend was observed in November. Therefore the increase in activity for November, which has the highest activity, is due to an increase in transplants utilising kidneys from both living and deceased organ donors. This contrasts with August, which had the lowest activity, and is solely explained by a fall in living donor activity.

**Incidence for brain injury donors fluctuates over the year**

For peer review only

Donation from cerebrovascular and hypoxic brain injury accounted for 78.8% of all deceased donor transplants, therefore we hypothesised that the spike in deceased donor transplants is due to an increase in donors from these categories during November and December. Cerebrovascular deaths, as a cause of donor death, were significantly increased in November (12% increase from the mean, $p<0.01$) (fig. 2a). Donations from donors who had experienced a hypoxic brain injury tended to increase during this period with the highest spike being in December (25% increase from the mean, $p=0.15$) (fig 2b). When these results are combined, they mirror the fluctuations seen for all deceased organ transplant activity (fig. 2c) with significant elevations in transplant activity during November and December ($p<0.01$).

**Seasonal Variance in the incidence of donors after infection**

We also noted that other causes of donor death significantly varied over the year (table 1). Interestingly, this variance was mainly observed in donations after brain death (table 2) rather than circulatory death (table 3). Donations after road traffic accidents (fig. 3a) were higher during late spring, summer and early autumn (April-September) compared to other times of the year. Donations from donors dying from infective causes peaked in the winter months and declined in the summer (fig3b) this relationship could be explained by a cosine curve (fig 3b, $p<0.05$) suggesting a potential underlying seasonal oscillation.

**The effect of healthcare capacity on transplant activity**

The U.K. healthcare system routinely works at high capacity[21], potentially resulting in reduced transplantation rates due to bed and staffing shortages. We therefore examined whether the reproducible monthly changes in transplant activity could be

For peer review only

attributed to a lack of capacity in the healthcare system. Although a large number 28,789 of deceased donor offers were declined for transplantation over the study period only a small fraction of these were due to lack of capacity (n=480 mean 1.22% (±0.22%SD).

For peer review only

### *Discussion*

The National Health Service in the U.K. is coping with increased pressures especially in winter[21]. This study reveals that UK kidney transplant activity peaks in November and December, at the beginning of winter, in contrast with a number of European[18 22] and North American centres[16 17]. This unexpected finding is due to increased living and deceased donation. Although detailed causal analysis was beyond the scope of the study, the increases in deceased donation could be attributed to both increases in cerebrovascular and hypoxic brain events. This is consistent with findings from previous studies studying the incidence of these events both in the UK[9] and other northern hemisphere countries[11 12].

The winter surge in transplant activity has important implications for the U.K. health system (NHS). During the UK winter there is also a surge in emergency admissions to hospitals, placing the system under significant strain, sometimes resulting in cancellation of elective and semi-elective operations for up to several months[23]. It was therefore reassuring that we found at the national level no solid evidence of transplant surgery utilising deceased donors being cancelled during the winter months. Despite this however it is important that individual transplant departments plan for this predictable and reproducible surge in transplant activity making sure their own activity is not affected. We are confident that this surge is likely to continue into the future as two different statistical tests (chi-square and GEE) produced similar results. This is especially important as UK transplant activity is likely to increase due to recent changes in legislation.

For peer review only

Certain limitations should be noted when interpreting the results of this study. We were not able to investigate whether healthcare capacity influenced the number of living donations since this data was not recorded. Since this was a retrospective study there is always the potential for inherent bias, although the large dataset will minimise this effect. Finally, each centre is responsible for submitting their own data, and therefore could not independently verify this data.

Our study clearly shows that UK renal transplant activity increases in the winter in contrast to previous studies investigating seasonal transplant activity in other countries. This could have implications for the UK health system since winter is when the British health system is placed under maximal strain. This seasonal variation should therefore be considered for any future planning especially with the potential impact of the opt out legislation and strategies to increase organ donation and transplantation.

**Authorship:** AT and JB conceived the study. AT, JB and ML obtained and analysed the data. RM did the statistical analysis. KP, JW, HD, DD, DWR, HD, AA substantially contributed to the interpretation of the data. AT, JB and AA drafted the manuscript, with the other authors revising it critically. .

**Conflicts of Interest:** The authors declare no conflicts of interest

**Funding:** JB holds a MRC clinician scientist award (MR/L006499/1). DWR holds a Wellcome Trust investigator award (107851/Z/15/Z) and a Medical Research Council grant (MR/P023576/1). HD is supported by an Asthma UK Senior Clinical Academic Development Award (AUK-SCAD-2013-229).

**Acknowledgement:** The authors would like to acknowledge Phil Foden for his contribution to the statistically analysis of the results, the NHSBT and U.K. renal transplant centers for collecting and providing the data. We would also like to acknowledge the NIHR Manchester Biomedical Research Centre who supported this work

For peer review only

## References

1. Transplant NBa. Organ Donation and Transplantation Activity report 2017/18, 2018.
2. Pyart R, Wong E, Sharples E, et al. Chapter 3 Demographic and Biochemistry Profile of Kidney Transplant Recipients in the UK in 2016. *Nephron* 2018;139 Suppl 1:75-104. doi: 10.1159/000490961 [published Online First: 2018/07/11]
3. Steenkamp R, Pyart R, Fraser S. Chapter 5 Survival and Cause of Death in UK Adult Patients on Renal Replacement Therapy in 2016. *Nephron* 2018;139 Suppl 1:117-50. doi: 10.1159/000490963 [published Online First: 2018/07/11]
4. Scriven DN. Capacity issues need to be tackled by looking at all of acute provision. *British Journal of Hospital Medicine* 2018;79(6):304-05. doi: 10.12968/hmed.2018.79.6.304
5. Rolden HJ, Rohling JH, van Bodegom D, et al. Seasonal Variation in Mortality, Medical Care Expenditure and Institutionalization in Older People: Evidence from a Dutch Cohort of Older Health Insurance Clients. *PloS one* 2015;10(11):e0143154. doi: 10.1371/journal.pone.0143154 [published Online First: 2015/11/17]
6. Paules C, Subbarao K. Influenza. *Lancet (London, England)* 2017 doi: 10.1016/s0140-6736(17)30129-0 [published Online First: 2017/03/18]
7. Anlar O, Tombul T, Unal O, et al. Seasonal and environmental temperature variation in the occurrence of ischemic strokes and intracerebral hemorrhages in a Turkish adult population. *The International journal of neuroscience* 2002;112(8):959-63. [published Online First: 2002/11/27]
8. de Steenhuijsen Piters WA, Algra A, van den Broek MF, et al. Seasonal and meteorological determinants of aneurysmal subarachnoid hemorrhage: a systematic review and meta-analysis. *Journal of neurology* 2013;260(2):614-9. doi: 10.1007/s00415-012-6687-z [published Online First: 2012/10/17]
9. Haberman S, Capildeo R, Rose FC. The seasonal variation in mortality from cerebrovascular disease. *Journal of the neurological sciences* 1981;52(1):25-36. [published Online First: 1981/10/01]
10. Mao Y, Schnytzer Y, Busija L, et al. "MOONSTROKE": Lunar patterns of stroke occurrence combined with circadian and seasonal rhythmicity--A hospital based study. *Chronobiology international* 2015;32(7):881-8. doi: 10.3109/07420528.2015.1049614 [published Online First: 2015/07/15]
11. Sipila JO, Ruuskanen JO, Kauko T, et al. Seasonality of stroke in Finland. *Annals of medicine* 2017;49(4):310-18. doi: 10.1080/07853890.2016.1254350 [published Online First: 2016/10/28]
12. Telman G, Sviri GE, Sprecher E, et al. Seasonal variation in spontaneous intracerebral hemorrhage in northern Israel. *Chronobiology international* 2017;34(5):563-70. doi: 10.1080/07420528.2016.1278223 [published Online First: 2017/02/06]
13. Yang L, Li L, Lewington S, et al. Outdoor temperature, blood pressure, and cardiovascular disease mortality among 23 000 individuals with diagnosed cardiovascular diseases from China. *European heart journal* 2015;36(19):1178-85. doi: 10.1093/eurheartj/ehv023 [published Online First: 2015/02/19]
14. Stevenson TJ, Visser ME, Arnold W, et al. Disrupted seasonal biology impacts health, food security and ecosystems. *Proceedings Biological sciences*

For peer review only

2015;282(1817):20151453. doi: 10.1098/rspb.2015.1453 [published Online First: 2015/10/16]
15. Kalkstein AJ. Regional similarities in seasonal mortality across the United States: an examination of 28 metropolitan statistical areas. *PloS one* 2013;8(5):e63971. doi: 10.1371/journal.pone.0063971 [published Online First: 2013/06/05]
16. Rosendale JD. Organ donation in the United States: 1988-2003. *Clinical transplants* 2004:41-50. [published Online First: 2006/05/18]
17. Grodin JL, Ayers CR, Thibodeau JT, et al. Variation of heart transplant rates in the United States during holidays. *Clinical transplantation* 2014;28(8):877-82. doi: 10.1111/ctr.12396 [published Online First: 2014/06/17]
18. Santori G, Fontana I, Bertocchi M, et al. A time series analysis performed on a 25-year period of kidney transplantation activity in a single center. *Transplantation proceedings* 2010;42(4):1080-3. doi: 10.1016/j.transproceed.2010.03.036 [published Online First: 2010/06/11]
19. Liang K-Y, Zeger SL. Longitudinal data analysis using generalized linear models. *Biometrika* 1986;73(1):13-22. doi: 10.1093/biomet/73.1.13
20. Menard S. Applied Logistic Regression Analysis. 2nd ed. USA: Sage 2002.
21. Iacobucci G. NHS "winter" pressure could become norm in summer, experts warn. *BMJ (Clinical research ed)* 2016;352:i905. doi: 10.1136/bmj.i905 [published Online First: 2016/02/14]
22. Leal J, Caballero F, Garcia-Sousa S, et al. Distribution of organ donors on a weekly and annual basis according to cause of death. *Transplantation proceedings* 1999;31(6):2602-3. [published Online First: 1999/09/29]
23. Iacobucci G. NHS cancels planned surgery and outpatient appointments in response to winter crisis. *BMJ (Clinical research ed)* 2018;360 doi: 10.1136/bmj.k19

For peer review only

# Tables

***Table 1***

| **Cause of death in the Donor** | **n** | **p value for whether donation varied across the year** |
|---|---|---|
| Intracranial / Cerebrovascular causes | 9452 | *<0.01* |
| Hypoxic brain damage | 2448 | *<0.01* |
| Trauma | 1532 | *<0.01* |
| Infective Causes | 367 | *<0.01* |
| Cardiovascular | 237 | 0.13 |
| Respiratory / Pulmonary causes | 192 | 0.17 |
| Brain Tumors | 180 | 0.14 |
| Poisoning / Drug overdose | 55 | 0.03 |
| Organ Failure (Various Causes) | 53 | *<0.01* |
| Other / Unknown | 578 | *0.01* |

**Table 1: Seasonal variation in kidney transplants utilising organs from deceased donors.** All deceased donations over a 10-year period were split into groups according to aetiology, defined by NHSBT. The incidence for the majority (7/10) of causes varied significantly during the year.

For peer review only

Table 2:

| **Cause of death in the Donor** | **n** | **p value for whether donation varied across the year** |
|---|---|---|
| Intracranial / Cerebrovascular causes | 6985 | <0.01 |
| Hypoxic brain damage | 1232 | <0.01 |
| Trauma | 970 | <0.01 |
| Infective Causes | 260 | <0.01 |
| Cardiovascular | 71 | <0.01 |
| Respiratory / Pulmonary causes | 7 | 0.18 |
| Brain Tumors | 149 | 0.57 |
| Poisoning / Drug overdose | 26 | <0.01 |
| Other / Unknown | 350 | 0.21 |

**Table 2: Seasonal variation in kidney transplants utilising organs from donors after brain death.** All donations after brain death over a 10-year period were split into groups according to aetiology, defined by NHSBT. The incidence for the majority (6/9) of causes varied significantly during the year.

For peer review only

Table 3:

| **Cause of death in the Donor** | **n** | **p value for whether donation varied across the year** |
|---|---|---|
| Intracranial / Cerebrovascular causes | | |
| Hypoxic brain damage | 1216 | 0.159 |
| Trauma | 562 | <0.01 |
| Infective Causes | 107 | 0.05 |
| Cardiovascular | 166 | 0.88 |
| Respiratory / Pulmonary causes | 185 | 0.25 |
| Brain Tumors | 31 | 0.07 |
| Poisoning / Drug overdose | 29 | <0.01 |
| Other / Unknown | 228 | <0.01 |

**Table 2: Seasonal variation in kidney transplants utilising organs from donors after circulatory death.** All donations after circulatory death over a 10-year period were split into groups according to aetiology, defined by NHSBT. The incidence for a minority (3/9) of causes varied significantly during the year.

For peer review only

# Figure legends

**Figure 1**: **Changes in transplant activity during the year. (a)** The number of UK renal transplants per month varied significantly during the year ($p<0.01$) peaking in November. **(b)** Transplants arising from living donors varied significantly throughout the year peaking in October and November **(c)** Transplant activity utilising deceased donors varied significantly over the year peaking on December (*=$p<0.05$, **=$p<0.01$ Pearson residual)

**Figure 2: Changes in transplant activity utilizing donors after cerebrovascular events or hypoxic brain injury. (a)** Transplant activity using donors following cerebrovascular events changed throughout the year increasing in November. **(b)** Transplant activity using donors with hypoxic brain damage also increased in activity at the end of the year (November and December). **(c)** By combining both these causes the graph mirrors the fluctuation seen in transplant activity for all deceased donors (shown in fig 1c). (*=$p<0.05$, **=$p<0.01$ Pearson residual)

**Figure 3: Seasonal variation in transplant activity arising after infection or trauma. (a)** Transplant activity using donors following traumatic incidents increased in the summer **(b)** as opposed to transplant activity utilising donors following infection which oscillated in a sinusoidal manner peaking in the winter. (*=$p<0.05$, **=$p<0.01$ Pearson residual, best fit line for infection is a cosine ($P<0.05$))

For peer review only

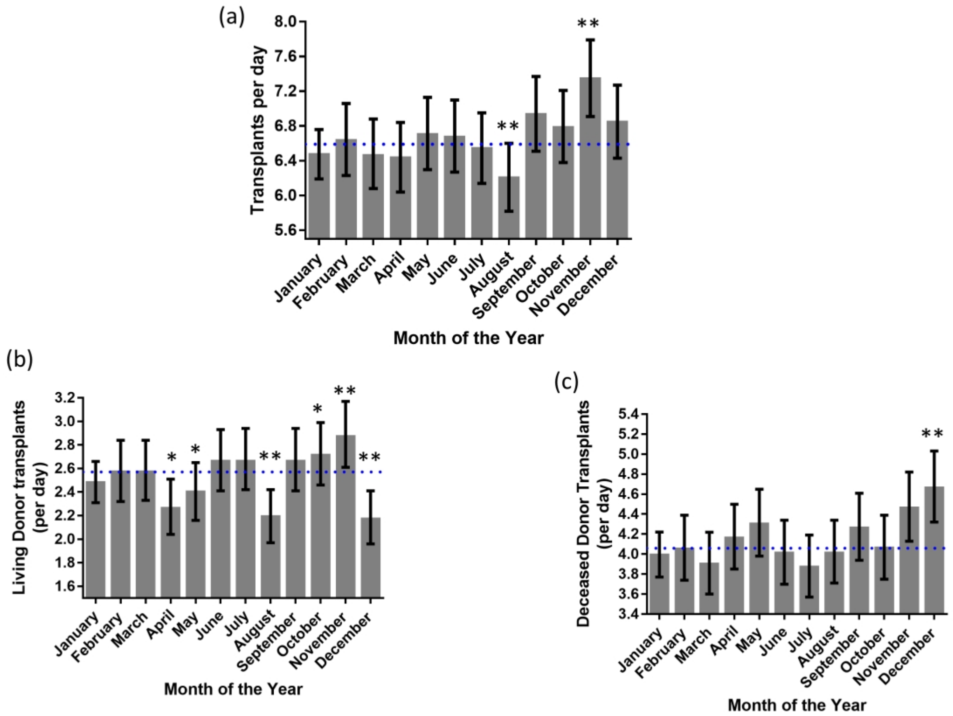


Figure 1: Changes in transplant activity during the year. (a) The number of UK renal transplants per month varied significantly during the year (p<0.01) peaking in November. (b) Transplants arising from living donors varied significantly throughout the year peaking in October and November (c) Transplant activity utilising deceased donors varied significantly over the year peaking on December (*=p<0.05, **=p<0.01 Pearson residual)

254x190mm (300 x 300 DPI)

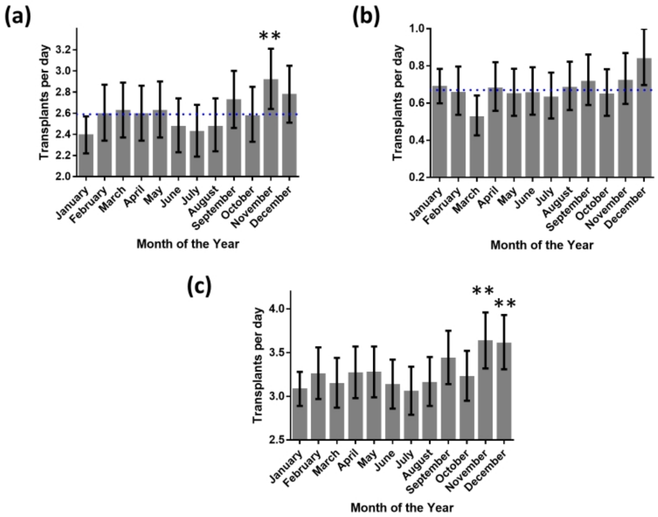


Figure 2: Changes in transplant activity utilizing donors after cerebrovascular events or hypoxic brain injury. (a) Transplant activity using donors following cerebrovascular events changed throughout the year increasing in November. (b) Transplant activity using donors with hypoxic brain damage also increased in activity at the end of the year (November and December). (c) By combining both these causes the graph mirrors the fluctuation seen in transplant activity for all deceased donors (shown in fig 1c). (*=p<0.05, **=p<0.01 Pearson residual)

254x190mm (300 x 300 DPI)

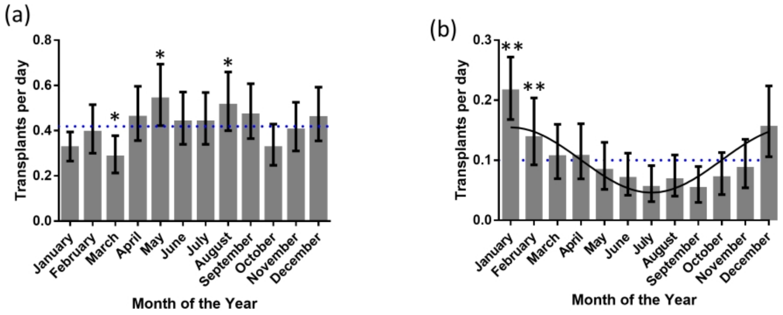


Figure 3: Seasonal variation in transplant activity arising after infection or trauma. (a) Transplant activity using donors following traumatic incidents increased in the summer (b) as opposed to transplant activity utilising donors following infection which oscillated in a sinusoidal manner peaking in the winter. (*=p<0.05, **=p<0.01 Pearson residual, best fit line for infection is a cosine (P<0.05))

254x190mm (300 x 300 DPI)

# Reporting checklist for cohort study.

Based on the STROBE cohort guidelines.

## Instructions to authors

Complete this checklist by entering the page numbers from your manuscript where readers will find each of the items listed below.

Your article may not currently address all the items on the checklist. Please modify your text to include the missing information. If you are certain that an item does not apply, please write "n/a" and provide a short explanation.

Upload your completed checklist as an extra file when you submit to a journal.

In your methods section, say that you used the STROBE cohort reporting guidelines, and cite them as:

von Elm E, Altman DG, Egger M, Pocock SJ, Gotzsche PC, Vandenbroucke JP. The Strengthening the Reporting of Observational Studies in Epidemiology (STROBE) Statement: guidelines for reporting observational studies.

| | | Reporting Item | Page Number |
|---|---|---|---|
| Title | #1a | Indicate the study’s design with a commonly used term in the title or the abstract | 1 |
| Abstract | #1b | Provide in the abstract an informative and balanced summary of what was done and what was found | 3 |
| Background / rationale | #2 | Explain the scientific background and rationale for the investigation being reported | 4 |
| Objectives | #3 | State specific objectives, including any prespecified hypotheses | 4 |
| Study design | #4 | Present key elements of study design early in the paper | 6 |
| Setting | #5 | Describe the setting, locations, and relevant dates, including periods of recruitment, exposure, follow-up, and data collection | 6 |
| Eligibility criteria | #6a | Give the eligibility criteria, and the sources and methods of selection of participants. Describe methods of follow-up. | 6 |
| | #6b | For matched studies, give matching criteria and number of exposed and | n/a |

For peer review only

| | | | |
|---|---|---|---|
| | | unexposed | |
| Variables | #7 | Clearly define all outcomes, exposures, predictors, potential confounders, and effect modifiers. Give diagnostic criteria, if applicable | 6 and 7 |
| Data sources / measurement | #8 | For each variable of interest give sources of data and details of methods of assessment (measurement). Describe comparability of assessment methods if there is more than one group. Give information separately for for exposed and unexposed groups if applicable. | 6 |
| Bias | #9 | Describe any efforts to address potential sources of bias | n/a |
| Study size | #10 | Explain how the study size was arrived at | 6 |
| Quantitative variables | #11 | Explain how quantitative variables were handled in the analyses. If applicable, describe which groupings were chosen, and why | 6 and 7 |
| Statistical methods | #12a | Describe all statistical methods, including those used to control for confounding | 7 |
| | #12b | Describe any methods used to examine subgroups and interactions | 7 |
| | #12c | Explain how missing data were addressed | n/a |
| | #12d | If applicable, explain how loss to follow-up was addressed | n/a |
| | #12e | Describe any sensitivity analyses | n/a |
| Participants | #13a | Report numbers of individuals at each stage of study—eg numbers potentially eligible, examined for eligibility, confirmed eligible, included in the study, completing follow-up, and analysed. Give information separately for for exposed and unexposed groups if applicable. | n/a |
| | #13b | Give reasons for non-participation at each stage | n/a |
| | #13c | Consider use of a flow diagram | n/a |
| Descriptive data | #14a | Give characteristics of study participants (eg demographic, clinical, social) and information on exposures and potential confounders. Give information separately for exposed and unexposed groups if applicable. | 10 |
| | #14b | Indicate number of participants with missing data for each variable of interest | 10 |
| | #14c | Summarise follow-up time (eg, average and total amount) | n/a |

For peer review only

| | | | |
|---|---|---|---|
| Outcome data | #15 | Report numbers of outcome events or summary measures over time. Give information separately for exposed and unexposed groups if applicable. | 10 |
| Main results | #16a | Give unadjusted estimates and, if applicable, confounder-adjusted estimates and their precision (eg, 95% confidence interval). Make clear which confounders were adjusted for and why they were included | 10-12 |
| | #16b | Report category boundaries when continuous variables were categorized | n/a |
| | #16c | If relevant, consider translating estimates of relative risk into absolute risk for a meaningful time period | n/a |
| Other analyses | #17 | Report other analyses done—e.g., analyses of subgroups and interactions, and sensitivity analyses | 10-12 |
| Key results | #18 | Summarise key results with reference to study objectives | 13-14 |
| Limitations | #19 | Discuss limitations of the study, taking into account sources of potential bias or imprecision. Discuss both direction and magnitude of any potential bias. | 13-14 |
| Interpretation | #20 | Give a cautious overall interpretation considering objectives, limitations, multiplicity of analyses, results from similar studies, and other relevant evidence. | 13-14 |
| Generalisability | #21 | Discuss the generalisability (external validity) of the study results | 13-14 |
| Funding | #22 | Give the source of funding and the role of the funders for the present study and, if applicable, for the original study on which the present article is based | 1 |

The STROBE checklist is distributed under the terms of the Creative Commons Attribution License CC-BY. This checklist was completed on 30. November 2018 using http://www.goodreports.org/, a tool made by the EQUATOR Network in collaboration with Penelope.ai

For peer review only

BMJ Open

# Monthly variance in United Kingdom renal transplantation activity: a national retrospective cohort study



| | |
|---|---|
| Journal: | *BMJ Open* |
| Manuscript ID | bmjopen-2018-028786.R2 |
| Article Type: | Research |
| Date Submitted by the Author: | 14-Jul-2019 |




| | |
|---|---|
| Complete List of Authors: | Lowe, Marcus; Manchester Foundation Trust, Transplantation Laboratory<br>Maidstone, Robert; University of Manchester, Faculty of Biology, Medicine and Health; University of Oxford, Oxford Centre for Diabetes, Endocrinology and Metabolism<br>Poulton, Kay; Manchester University Foundation NHS trust, Transplantation Laboratory<br>Worthington, Judith; Manchester University Foundation NHS trust, Transplantation Laboratory<br>Durrington, Hannah J.; University of Manchester, Faculty of Biology, Medicine and Health; Manchester University Foundation NHS trust, Department of Respiratory Medicine<br>Ray, David; John Radcliffe Hospital, NIHR Oxford Biomedical Research Centre; University of Oxford, Oxford Centre for Diabetes, Endocrinology and Metabolism<br>van Dellen, David; University of Manchester, Faculty of Biology, Medicine and Health; Manchester University Foundation NHS trust, Transplant and Endocrine Surgery<br>Asderakis, Argiris; University Hospital of Wales, Cardiff Transplant Unit<br>Blaikley, John; University of Manchester Faculty of Biology, Medicine and Health, ; Manchester University Foundation NHS trust, Medicine<br>Augustine, Titus; University of Manchester, Faculty of Biology, Medicine and Health; Manchester University Foundation NHS trust, Transplant and Endocrine Surgery |




| | |
|---|---|
| <b>Primary Subject Heading</b>: | Renal medicine |
| Secondary Subject Heading: | Renal medicine, Health policy, Urology |
| Keywords: | Renal transplantation < NEPHROLOGY, Seasonal, Healthcare planning |
| | |


SCHOLARONE™
Manuscripts

**Title** Monthly variance in United Kingdom renal transplantation activity: a national retrospective cohort study

***Authors***

Marcus P. Lowe[1], Robert Maidstone[2,3], Kay V. Poulton[1], Judith E. Worthington[1], Hannah Durrington[2,4], David W. Ray[3,5], David van Dellen[2,6], Argiris Asderakis[7], John Blaikley[2,8†], Titus Augustine[2,6†]

***Affiliations***

[1]Transplantation Laboratory, Manchester Royal Infirmary, Manchester University NHS Foundation Trust

[2]Faculty of Biology, Medicine and Health, University of Manchester, Manchester, UK

[3]Oxford Centre for Diabetes, Endocrinology and Metabolism, University of Oxford, OX37LE

[4]Department of Respiratory Medicine, Manchester University NHS Foundation Trust, Manchester, UK

[5]NIHR Oxford Biomedical Research Centre, John Radcliffe Hospital OX3 9DU

[6]Transplant and Endocrine Surgery, Manchester University NHS Foundation Trust, Manchester, UK

[7]Cardiff Transplant Unit, University Hospital of Wales, Cardiff

[8]Department of Cardiothoracic Medicine and Transplantation, Manchester University NHS Foundation Trust

**†= joint corresponding authors**

**Correspondence can be addressed to: titus.augustine@mft.nhs.uk or john.blaikley@manchester.ac.uk**

For peer review only

Keywords: Renal transplantation, Seasonal Variation, Healthcare Planning

Word Count: 1,918

For peer review only

## Abstract

**Objective:** To identify whether renal transplant activity varies in a reproducible manner across the year

**Design:** Retrospective cohort study using NHS blood and transplant (NHSBT) data

**Setting:** All renal transplant centers in the UK

**Participants:** 24,270 patients who underwent renal transplantation between 2005 and 2014

**Primary outcome:** Monthly transplant activity was analysed to see if transplant activity showed variation during the year

**Secondary Outcome:** The number of organs rejected due to healthcare capacity was analysed to see if this affected transplantation rates.

**Results:** Analysis of national transplant data revealed a reproducible yearly variance in transplant activity. This activity increased in late autumn and early winter (p=0.05) and could be attributed to increased rates of living (October-November) and deceased organ donation (November, December). An increase in deceased donation was attributed to a rise in donors following cerebrovascular accidents and hypoxic brain injury. Other causes of death (infections and road traffic accidents) were more seasonal in nature peaking in the winter or summer respectively. Only 1.4% of transplants to intended recipients were redirected due to a lack of healthcare capacity, suggesting that capacity pressures in the NHS did not significantly affect transplant activity.

**Conclusion:** UK renal transplant activity peaks in late autumn/ winter in contrast to other countries. Currently healthcare capacity, though under strain, does not affect transplant activity; however this may change if transplantation activity increases in line


For peer review only

with national strategies as the spike in transplant activity coincides with peak activity in the national healthcare system.

For peer review only

**Strengths and limitations of the study**

- The study was a 10-year retrospective study involving all the registered renal transplant recipients in the UK over this time period.
- The national transplant database is filled using data submitted by each transplant center therefore, the data has not been independently corroborated.
- A minority of patients will have refused to be enrolled in the national database potentially affecting our findings.
- The database is used to audit transplant provision and outcomes in the UK therefore it was not set up specifically for this research project

For peer review only

# Introduction

When organ donors and families of potential donors consent to donation, they make a very valuable gift which is life changing for the recipient. Despite recent improvements in the United Kingdom (UK)[1] and other countries regarding access to transplantation, demand for renal transplantation still exceeds the number of available donors[2]. This results in increased patient mortality and morbidity[3]. Since many potential donors cannot be utilised for various reasons, it is vital that these reasons are minimised so that every potential organ for transplant is utilised for the primary intended recipient. One of the reasons transplantation does not proceed is due to a lack of clinical capacity. Therefore healthcare planning plays a key role in ensuring that sufficient capacity exists so that all transplants are utilised for the primary intended recipients. This could be potentially difficult as the NHS often works near or at maximum capacity[4], especially in winter months.

Renal transplantation uses both living and deceased donors. Human mortality rates are known to oscillate in a seasonal manner for some diseases[5]. This can be attributed to the effects of endogenous seasonal rhythms and climatic factors on human performance and activity patterns. In the U.K. seasonal variations are commonly observed for infectious diseases such as influenza[6], cerebrovascular disease[7-12] and myocardial infarctions[13]. Several studies have recently shown how understanding these oscillations are crucial for planning and delivering optimum health care delivery[14]. Despite the USA covering many different climate zones, cerebrovascular accidents in the USA peak in the winter[15], in a similar pattern to the UK[7-12]. Surprisingly despite these deaths being a leading cause of organ donation transplant activity in the USA is lowest in the winter months for both renal[16] and heart transplantation[17]. A

For peer review only - http://bmjopen.bmj.com/site/about/guidelines.xhtml

similar pattern is also seen in Italy[18], suggesting this seasonal pattern is conserved in other countries.

To the best of our knowledge seasonal fluctuations in transplant activity have not been investigated in the UK. We therefore examined the national cohort over a 10-year period to establish both seasonal fluctuations and whether healthcare capacity influences activity.

For peer review only

# Methods

All UK renal adult transplants, performed between 2005 and 2014 to recipients over 18 years old, were included in the study. Data was provided by NHSBT (NHS Blood and Transplant) who maintain a comprehensive national database on the 24 adult kidney transplant centres in the United Kingdom. This data was combined with data collected on donor activity, provided by the National Organ Retrieval Service (NORS). Deceased donation is categorised into donation after brain death (DBD) and donation after circulatory death DCD). All donor deaths are further classified according to the cause of mortality. "Cerebrovascular event" was defined as death due to intracranial haemorrhage, intracranial thrombus or unclassified intracranial event. "Infection" was defined as death due to meningitis, septicaemia, pneumonia or unclassified infection. Deceased donor transplants form about two thirds of all UK kidney transplant activity. The other one third of kidney transplants are transplants from living donors. Living donor transplants are logistically different as they are planned and scheduled elective cases depending on several factors which can usually be controlled. Deceased donor transplants on the other hand are unplanned occurring when organs become available after death of a suitable donor. Recipients are then allocated organs according to agreed national allocation criteria.

Data was analysed according to the month and year the transplant occurred. Donation details were analysed in a similar manner. The observed data was compared to the expected transplant activity calculated by measuring the total number of transplants divided by the number of days in the same year.

Data was also analysed according to organs offered to different centres for named patients and declined, for various reasons by the centre. Organs are declined for various clinical reasons and, anecdotally, rarely due to capacity and peaks of activity.

For peer review only

Capacity issues for the purposes of this paper were sub classified into different categorical values (centre already transplanting, no beds, no staff, no theatre, no time).

### *Statistical methods*

Generalised linear models (GLMs), with a Poisson link function were used to model the effect that month of the year had on our data. Offset variables were used to account for the slight differences in number of days per month. To account for the unknown correlation structure in our data, generalised estimating equations (GEEs) per year were used to estimate the GLM parameters[19].

Chi-squared goodness of fit tests assessed whether the observed number of transplants per month differed from the expected value if there was no seasonal variability. When this test was significant (i.e. the expected values were significantly different to the observed values), Pearson residuals were used to identify the particular months which caused this mismatch. Residuals that are greater than 2 in magnitude suggest some degree of lack of fit[20].

Sine and cosine curves were fitted to the transplant data to investigate whether repeating patterns occurred across the months. All analyses were performed using SPSS 22 (IBM corp)

**Patient and Public Involvement:**

Patients and Public were not involved in the design or analysis of data for this study

**Ethics:**

Data was collected by NHSBT and analysed after obtaining approval from them in accordance with their policies. Since the data was fully anonymized no ethical permission was sought.

**Data Availability:**

For peer review only

Raw data will be available from the corresponding author after permission has been obtained from NHSBT. The statistical code has been uploaded onto a data repository DOI: 10.17632/48nxwvcfnh.1

For peer review only

# Results

During the ten-year study period 24,270 adult kidney transplants were performed in the UK. 15,094 (62%) were from deceased donors and 9,166 (38%) were from living donors

**Kidney Transplant activity changes in a consistent manner throughout the year**

Kidney Transplant activity varied by 17.1% within a year ($p<0.01$, chi-square, fig 1a). Transplant activity was highest in November (11% increase from mean, $p<0.01$) and lowest in August (6% decrease from mean, $p<0.01$). Investigating seasonal variation, transplant activity increased in the autumn compared to spring and summer ($p<0.05$ one-way ANOVA); these patterns were consistent every year during the 10 year study period ($p<0.01$ GEE).

Kidney transplantation utilises both living and deceased donors. Our dataset was therefore examined to see if one of these types was responsible for the variance in activity. Transplant activity utilising organs from living donors (fig. 1b) significantly increased in October and November and decreased in December, April, May and August. Transplant activity utilising organs from deceased donors (fig. 1c) increased in December and a similar trend was observed in November. Therefore the increase in activity for November, which has the highest activity, is due to an increase in transplants utilising kidneys from both living and deceased organ donors. This contrasts with August, which had the lowest activity, and is solely explained by a fall in living donor activity.

**Incidence for brain injury donors fluctuates over the year**

For peer review only

Donation from cerebrovascular and hypoxic brain injury accounted for 78.8% of all deceased donor transplants, therefore we hypothesised that the spike in deceased donor transplants is due to an increase in donors from these categories during November and December. Cerebrovascular deaths, as a cause of donor death, were significantly increased in November (12% increase from the mean, $p<0.01$) (fig. 2a). Donations from donors who had experienced a hypoxic brain injury tended to increase during this period with the highest spike being in December (25% increase from the mean, $p=0.15$) (fig 2b). When these results are combined, they mirror the fluctuations seen for all deceased organ transplant activity (fig. 2c) with significant elevations in transplant activity during November and December ($p<0.01$).

**Seasonal Variance in the incidence of donors after infection**

We also noted that other causes of donor death significantly varied over the year (table 1). Interestingly, this variance was mainly observed in donations after brain death (table 2) rather than circulatory death (table 3). Donations after road traffic accidents (fig. 3a) were higher during late spring, summer and early autumn (April-September) compared to other times of the year. Donations from donors dying from infective causes peaked in the winter months and declined in the summer (fig3b) this relationship could be explained by a cosine curve (fig 3b, $p<0.05$) suggesting a potential underlying seasonal oscillation.

**The effect of healthcare capacity on transplant activity**

The U.K. healthcare system routinely works at high capacity[21], potentially resulting in reduced transplantation rates due to bed and staffing shortages. We therefore examined whether the reproducible monthly changes in transplant activity could be

For peer review only - http://bmjopen.bmj.com/site/about/guidelines.xhtml

attributed to a lack of capacity in the healthcare system. Although a large number 28,789 of deceased donor offers were declined for transplantation over the study period only a small fraction of these were due to lack of capacity (n=480 mean 1.22% (±0.22%SD).

For peer review only

### *Discussion*

The National Health Service in the U.K. is coping with increased pressures especially in winter[21]. This study reveals that UK kidney transplant activity peaks in November and December, at the beginning of winter, in contrast with a number of European[18 22] and North American centres[16 17]. This unexpected finding is due to variations in living and deceased donation. Although detailed causal analysis was beyond the scope of the study, the winter increases in deceased donation could be attributed to both increases in cerebrovascular and hypoxic brain events. This is consistent with findings from previous studies studying the incidence of these events both in the UK[9] and other northern hemisphere countries[11 12].

The winter surge in transplant activity has important implications for the U.K. health system (NHS). During the UK winter there is also a surge in emergency admissions to hospitals, placing the system under significant strain, sometimes resulting in cancellation of elective and semi-elective operations for up to several months[23]. It was therefore reassuring that we found at the national level no solid evidence of transplant surgery utilising deceased donors being cancelled during the winter months. Despite this however it is important that individual transplant departments plan for this predictable and reproducible surge in transplant activity making sure their own activity is not affected. We are confident that this surge is likely to continue into the future as two different statistical tests (chi-square and GEE) produced similar results. This is especially important as UK transplant activity is likely to increase due to recent changes in legislation.

For peer review only

Certain limitations should be noted when interpreting the results of this study. The donor rates reported in the paper are from utilised donations and therefore can be influenced by changes in donor conversion rates; the proportion of donor offers used in a transplant operation. This is unlikely however as these changes would have to be consistent occurring in the same way each year for ten years, furthermore the peak of deceased donation activity coincided with the epidemiological peaks for the underlying diseases[7-12]. Since this was a retrospective study there is always the potential for inherent bias, but since this is relatively large dataset it should minimise this effect. The data from each centre could not be independently verified, due to anonymisation in the dataset. Finally, the effect of healthcare capacity on living donations was not investigated, but this would be an important area to investigate in future research.

Our study clearly shows that UK renal transplant activity increases in the winter in contrast to previous studies investigating seasonal transplant activity in other countries. This could have implications for the UK health system since winter is when the British health system is placed under maximal strain. This seasonal variation should therefore be considered for any future planning especially with the potential impact of the opt out legislation and strategies to increase organ donation and transplantation.

**Authorship:** AT and JB conceived the study. AT, JB and ML obtained and analysed the data. RM did the statistical analysis. KP, JW, HD, DD, DWR, HD, AA substantially contributed to the interpretation of the data. AT, JB and AA drafted the manuscript, with the other authors revising it critically.

**Conflicts of Interest:** The authors declare no conflicts of interest

**Funding:** JB holds a MRC clinician scientist award (MR/L006499/1). DWR holds a Wellcome Trust investigator award (107851/Z/15/Z) and a Medical Research

Council grant (MR/P023576/1). HD is supported by an Asthma UK Senior Clinical Academic Development Award (AUK-SCAD-2013-229). JB and HD are partially supported by the Manchester NIHR biomedical research Centre

**Acknowledgement:** The authors would like to acknowledge Phil Foden for his contribution to the statistically analysis of the results, the NHSBT and U.K. renal transplant centers for collecting and providing the data.

For peer review only

## References


1. Transplant NBa. Organ Donation and Transplantation Activity report 2017/18, 2018.
2. Pyart R, Wong E, Sharples E, et al. Chapter 3 Demographic and Biochemistry Profile of Kidney Transplant Recipients in the UK in 2016. *Nephron* 2018;139 Suppl 1:75-104. doi: 10.1159/000490961 [published Online First: 2018/07/11]
3. Steenkamp R, Pyart R, Fraser S. Chapter 5 Survival and Cause of Death in UK Adult Patients on Renal Replacement Therapy in 2016. *Nephron* 2018;139 Suppl 1:117-50. doi: 10.1159/000490963 [published Online First: 2018/07/11]
4. Scriven DN. Capacity issues need to be tackled by looking at all of acute provision. *British Journal of Hospital Medicine* 2018;79(6):304-05. doi: 10.12968/hmed.2018.79.6.304
5. Rolden HJ, Rohling JH, van Bodegom D, et al. Seasonal Variation in Mortality, Medical Care Expenditure and Institutionalization in Older People: Evidence from a Dutch Cohort of Older Health Insurance Clients. *PloS one* 2015;10(11):e0143154. doi: 10.1371/journal.pone.0143154 [published Online First: 2015/11/17]
6. Paules C, Subbarao K. Influenza. *Lancet (London, England)* 2017 doi: 10.1016/s0140-6736(17)30129-0 [published Online First: 2017/03/18]
7. Anlar O, Tombul T, Unal O, et al. Seasonal and environmental temperature variation in the occurrence of ischemic strokes and intracerebral hemorrhages in a Turkish adult population. *The International journal of neuroscience* 2002;112(8):959-63. [published Online First: 2002/11/27]
8. de Steenhuijsen Piters WA, Algra A, van den Broek MF, et al. Seasonal and meteorological determinants of aneurysmal subarachnoid hemorrhage: a systematic review and meta-analysis. *Journal of neurology* 2013;260(2):614-9. doi: 10.1007/s00415-012-6687-z [published Online First: 2012/10/17]
9. Haberman S, Capildeo R, Rose FC. The seasonal variation in mortality from cerebrovascular disease. *Journal of the neurological sciences* 1981;52(1):25-36. [published Online First: 1981/10/01]
10. Mao Y, Schnytzer Y, Busija L, et al. "MOONSTROKE": Lunar patterns of stroke occurrence combined with circadian and seasonal rhythmicity--A hospital based study. *Chronobiology international* 2015;32(7):881-8. doi: 10.3109/07420528.2015.1049614 [published Online First: 2015/07/15]
11. Sipila JO, Ruuskanen JO, Kauko T, et al. Seasonality of stroke in Finland. *Annals of medicine* 2017;49(4):310-18. doi: 10.1080/07853890.2016.1254350 [published Online First: 2016/10/28]
12. Telman G, Sviri GE, Sprecher E, et al. Seasonal variation in spontaneous intracerebral hemorrhage in northern Israel. *Chronobiology international* 2017;34(5):563-70. doi: 10.1080/07420528.2016.1278223 [published Online First: 2017/02/06]
13. Yang L, Li L, Lewington S, et al. Outdoor temperature, blood pressure, and cardiovascular disease mortality among 23 000 individuals with diagnosed cardiovascular diseases from China. *European heart journal* 2015;36(19):1178-85. doi: 10.1093/eurheartj/ehv023 [published Online First: 2015/02/19]
14. Stevenson TJ, Visser ME, Arnold W, et al. Disrupted seasonal biology impacts health, food security and ecosystems. *Proceedings Biological sciences* 2015;282(1817):20151453. doi: 10.1098/rspb.2015.1453 [published Online First: 2015/10/16]


For peer review only

15. Kalkstein AJ. Regional similarities in seasonal mortality across the United States: an examination of 28 metropolitan statistical areas. *PloS one* 2013;8(5):e63971. doi: 10.1371/journal.pone.0063971 [published Online First: 2013/06/05]
16. Rosendale JD. Organ donation in the United States: 1988-2003. *Clinical transplants* 2004:41-50. [published Online First: 2006/05/18]
17. Grodin JL, Ayers CR, Thibodeau JT, et al. Variation of heart transplant rates in the United States during holidays. *Clinical transplantation* 2014;28(8):877-82. doi: 10.1111/ctr.12396 [published Online First: 2014/06/17]
18. Santori G, Fontana I, Bertocchi M, et al. A time series analysis performed on a 25-year period of kidney transplantation activity in a single center. *Transplantation proceedings* 2010;42(4):1080-3. doi: 10.1016/j.transproceed.2010.03.036 [published Online First: 2010/06/11]
19. Liang K-Y, Zeger SL. Longitudinal data analysis using generalized linear models. *Biometrika* 1986;73(1):13-22. doi: 10.1093/biomet/73.1.13
20. Menard S. Applied Logistic Regression Analysis. 2nd ed. USA: Sage 2002.
21. Iacobucci G. NHS "winter" pressure could become norm in summer, experts warn. *BMJ (Clinical research ed)* 2016;352:i905. doi: 10.1136/bmj.i905 [published Online First: 2016/02/14]
22. Leal J, Caballero F, Garcia-Sousa S, et al. Distribution of organ donors on a weekly and annual basis according to cause of death. *Transplantation proceedings* 1999;31(6):2602-3. [published Online First: 1999/09/29]
23. Iacobucci G. NHS cancels planned surgery and outpatient appointments in response to winter crisis. *BMJ (Clinical research ed)* 2018;360 doi: 10.1136/bmj.k19

For peer review only

# Tables

| **Cause of death in the Donor** | **n** | **p value for whether donation varied across the year** |
|---|---|---|
| Intracranial / Cerebrovascular causes | 9452 | *<0.01* |
| Hypoxic brain damage | 2448 | *<0.01* |
| Trauma | 1532 | *<0.01* |
| Infective Causes | 367 | *<0.01* |
| Cardiovascular | 237 | 0.13 |
| Respiratory / Pulmonary causes | 192 | 0.17 |
| Brain Tumors | 180 | 0.14 |
| Poisoning / Drug overdose | 55 | 0.03 |
| Organ Failure (Various Causes) | 53 | *<0.01* |
| Other / Unknown | 578 | *0.01* |

**Table 1: Seasonal variation in kidney transplants utilising organs from deceased donors.** All deceased donations over a 10-year period were split into groups according to aetiology, defined by NHSBT. The incidence for the majority (7/10) of causes varied significantly during the year.

For peer review only

| **Cause of death in the Donor** | **n** | **p value for whether donation varied across the year** |
|---|---|---|
| Intracranial / Cerebrovascular causes | 6985 | <0.01 |
| Hypoxic brain damage | 1232 | <0.01 |
| Trauma | 970 | <0.01 |
| Infective Causes | 260 | <0.01 |
| Cardiovascular | 71 | <0.01 |
| Respiratory / Pulmonary causes | 7 | 0.18 |
| Brain Tumors | 149 | 0.57 |
| Poisoning / Drug overdose | 26 | <0.01 |
| Other / Unknown | 350 | 0.21 |

**Table 2: Seasonal variation in kidney transplants utilising organs from donors after brain death.** All donations after brain death over a 10-year period were split into groups according to aetiology, defined by NHSBT. The incidence for the majority (6/9) of causes varied significantly during the year.

For peer review only

| **Cause of death in the Donor** | **n** | **p value for whether donation varied across the year** |
|---|---|---|
| Intracranial / Cerebrovascular causes | | |
| Hypoxic brain damage | 1216 | 0.159 |
| Trauma | 562 | <0.01 |
| Infective Causes | 107 | 0.05 |
| Cardiovascular | 166 | 0.88 |
| Respiratory / Pulmonary causes | 185 | 0.25 |
| Brain Tumors | 31 | 0.07 |
| Poisoning / Drug overdose | 29 | <0.01 |
| Other / Unknown | 228 | <0.01 |

**Table 3: Seasonal variation in kidney transplants utilising organs from donors after circulatory death.** All donations after circulatory death over a 10-year period were split into groups according to aetiology, defined by NHSBT. The incidence for a minority (3/9) of causes varied significantly during the year.

For peer review only

# Figure legends

**Figure 1**: **Changes in transplant activity during the year.** **(a)** The number of UK renal transplants per month varied significantly during the year ($p<0.01$) peaking in November. **(b)** Renal transplants arising from living donors varied significantly throughout the year peaking in October and November **(c)** Transplant activity utilising deceased donors varied significantly over the year peaking on December (*=$p<0.05$, **=$p<0.01$ Pearson residual, dotted line shows average activity)

**Figure 2: Changes in transplant activity utilizing donors after cerebrovascular events or hypoxic brain injury. (a)** Transplant activity utilising donors following cerebrovascular events peaks in November. **(b)** Transplant activity utilising donors with hypoxic brain damage also increased in activity at the end of the year (November and December). **(c)** By combining both these causes the graph mirrors the fluctuation seen in transplant activity for all deceased donors (shown in fig 1c). (*=$p<0.05$, **=$p<0.01$ Pearson residual, dotted line shows average activity)

**Figure 3: Seasonal variation in transplant activity arising after infection or trauma. (a)** Transplant activity using donors following traumatic incidents increased in the summer **(b)** as opposed to transplant activity utilising donors following infection which oscillated in a sinusoidal manner peaking in the winter. (*=$p<0.05$, **=$p<0.01$ Pearson residual, best fit line for infection is a cosine ($P<0.05$))

For peer review only

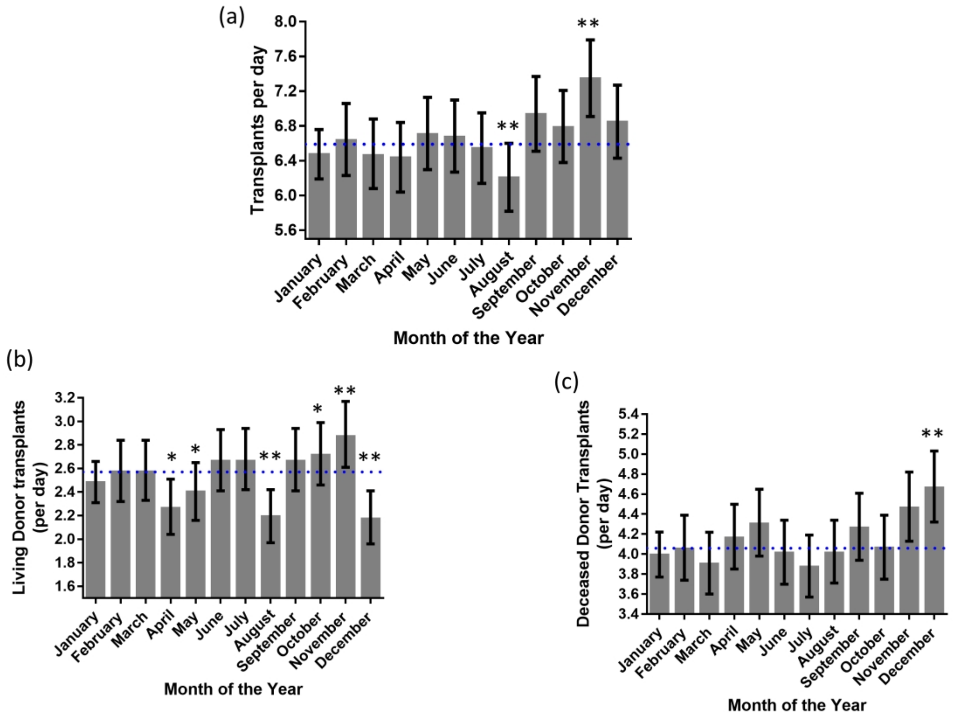


Figure 1: Changes in transplant activity during the year. (a) The number of UK renal transplants per month varied significantly during the year (p<0.01) peaking in November. (b) Renal transplants arising from living donors varied significantly throughout the year peaking in October and November (c) Transplant activity utilising deceased donors varied significantly over the year peaking on December (*=p<0.05, **=p<0.01 Pearson residual, dotted line shows average activity)

254x190mm (300 x 300 DPI)

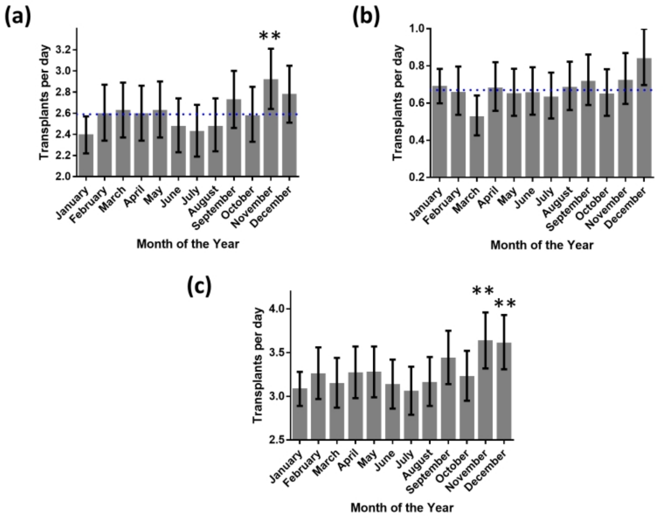


Figure 2: Changes in transplant activity utilizing donors after cerebrovascular events or hypoxic brain injury. (a) Transplant activity using donors following cerebrovascular events changed throughout the year increasing in November. (b) Transplant activity using donors with hypoxic brain damage also increased in activity at the end of the year (November and December). (c) By combining both these causes the graph mirrors the fluctuation seen in transplant activity for all deceased donors (shown in fig 1c). (*=p<0.05, **=p<0.01 Pearson residual)

254x190mm (300 x 300 DPI)

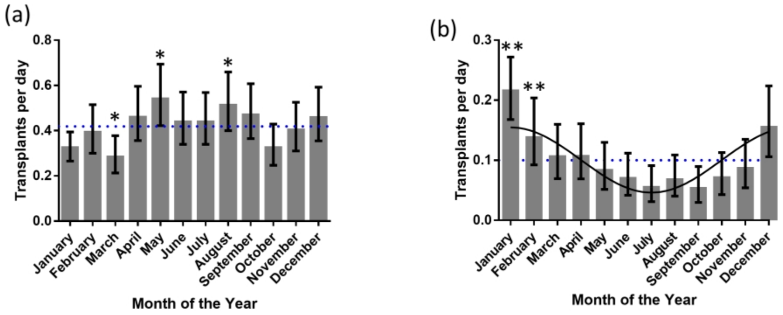


Figure 3: Seasonal variation in transplant activity arising after infection or trauma. (a) Transplant activity using donors following traumatic incidents increased in the summer (b) as opposed to transplant activity utilising donors following infection which oscillated in a sinusoidal manner peaking in the winter. (*=p<0.05, **=p<0.01 Pearson residual, best fit line for infection is a cosine (P<0.05))

254x190mm (300 x 300 DPI)
